# Draft Genome Sequence of the Boron-Tolerant and Moderately Halotolerant Bacterium *Gracilibacillus boraciitolerans* JCM 21714^T^

**DOI:** 10.1128/genomeA.00097-14

**Published:** 2014-02-20

**Authors:** Iftikhar Ahmed, Kenshiro Oshima, Wataru Suda, Keiko Kitamura, Toshiya Iida, Yoshihiro Ohmori, Toru Fujiwara, Masahira Hattori, Moriya Ohkuma

**Affiliations:** a Japan Collection of Microorganisms/Microbe Division, RIKEN BioResource Center, Tsukuba, Japan; b National Institute for Genomics and Advanced Biotechnology (NIGAB), National Agricultural Research Centre (NARC), Islamabad, Pakistan; c Laboratory of Plant Nutrition and Fertilizers, Department of Applied Biological Chemistry, Graduate School of Agricultural and Life Sciences, the University of Tokyo, Tokyo, Japan; d Centre for Omics and Bioinformatics, Graduate School of Frontier Sciences, the University of Tokyo, Kashiwa, Japan

## Abstract

*Gracilibacillus boraciitolerans* JCM 21714^T^ has been characterized as a highly boron-tolerant and moderately halotolerant bacterium. Here, we report the draft genome sequence of this strain. The genome sequence facilitates an understanding of the biochemical functions of boron and provides a base to identify the gene(s) involved in the boron tolerance mechanism of the strain.

## GENOME ANNOUNCEMENT

Boron (B) has been reported as an essential micronutrient for the optimum growth of plants (1) but is toxic to living cells when present at concentrations over a certain threshold. Previously, the physiological analysis of B tolerance in bacteria revealed a negative correlation between the degree of tolerance to high external B and the protoplasmic B concentrations (2). *Gracilibacillus boraciitolerans* was identified as highly boron-tolerant and moderately halotolerant bacterium, which can tolerate >450 mM B and up to 11% NaCl, respectively (3). This bacterium is Gram-positive, motile, rod-shaped, and endospore-forming, isolated from naturally boron-contaminated soil of the Hisarcik area in Kutahya Province, Turkey. The growth of this strain occurs at 16 to 37°C (optimum, 25 to 28°C) and pH 6.0 to 10.0 (optimum, 7.5 to 8.5) in tryptic soy broth without the addition of NaCl or boron.

The genome of *G. boraciitolerans* JCM 21714^T^ was sequenced using the Ion Torrent PGM system. A total of 558,321 quality-filtered reads were assembled into 88 contigs using Newbler version 2.8 (Roche) (70 contigs, >2,000 bp; longest, 619,400 bp; shortest, 638 bp), with an N_50_ length of 93,666 bp, which resulted in a draft genome sequence of 3,651,580 bp with 33.7× redundancy and a G+C content of 35.8%.

The draft genome of *G. boraciitolerans* JCM21714^T^ contains 4,450 coding sequences, single copies of the 16S rRNA and 23S rRNA genes, two copies of 5S rRNA genes, and 58 tRNAs genes, as predicted using the Rapid Annotations using Subsystems Technology (RAST) version 2.0 (4) and RNAmmer version 1.2 (5) servers. The RAST server predicted 44% of the annotatable open reading frames (ORFs) to encode known proteins. There were 415 subsystems identified in the genome, with the major subsystems represented by the genes involved in carbohydrate metabolism (395 genes), amino acids and derivatives (287 genes), protein metabolism (239 genes), and many others, including those involved in membrane transport (92 genes), stress response (90 genes), fatty acids, lipids, and isoprenoids (114 genes), dormancy and sporulation (114 genes), motility and chemotaxis (98 genes), cell wall and capsule (101 genes), iron acquisition and metabolism (4 genes), and virulence, disease, and defense (67 genes); however, no gene was detected for photosynthesis.

Our BLASTp search (*E* value, <1e − 5) indicated that the genome of *G. boraciitolerans* JCM 21714^T^ contains boron transport-related genes of yeast and plants: 2 orthologs of the Atr1p gene (6), 66 of the AtNIP5:1 gene (7, 8), 54 of the AtPIP1 gene (9), 30 of the Dur3p gene (10), and 17 of the Fps1 gene (10). There was no orthologous gene related to the Bor1 (11) or AtBor1 (12) genes, which are the most critical for boron transport in plants. A detailed comparative genome analysis will elucidate the genes involved in stress tolerance in boron, which might provide us with useful information on the application of a boron-tolerant bacterium in food and agricultural industries.

### Nucleotide sequence accession numbers.

The draft whole genome sequence of *G. boraciitolerans* JCM 21714^T^ has been deposited in DDBJ/EMBL/GenBank under accession no. BAVS01000001 to BAVS01000088.
